# In silico data analyses of recombinases GdDMC1A and GdDMC1B from *Giardia duodenalis*

**DOI:** 10.1016/j.dib.2016.08.031

**Published:** 2016-08-22

**Authors:** Ana Laura Torres-Huerta, Rosa María Martínez-Miguel, María Luisa Bazán-Tejeda, Rosa María Bermúdez-Cruz

**Affiliations:** Genetics and Molecular Biology Department, Centro de Investigación y de Estudios Avanzados del IPN, Av. Instituto Politécnico Nacional No. 2508, C.P. 07360 México D.F., Mexico

**Keywords:** In silico analyses, DMC1B and DMC1A, 3D structure prediction, DL50 for ionizing radiation

## Abstract

*Giardia duodenalis* is a worldwide protozoa known causing diarrhea in all vertebrates, humans among these. Homologous recombination is a mechanism that provides genomic stability. Two putative recombinases were identified in *G. duodenalis* genome: GdDMC1A and GdDMC1B. In this article, we describe the identification of conserved domains in GdDMC1A and GdDMC1B, such as: DNA binding domains (Helix-turn-helix motif, loops 1 and 2) and an ATPcap and Walker A and B motifs associated with ATP binding and hydrolysis, phylogenetic analyses among assemblages and three-dimensional structure modeling of these recombinases using bioinformatics tools. Also, experimental data is described about LD50 determination for ionizing radiation in trophozoites of *G. duodenalis*. Additionally, as recombinases, GdDMC1A and GdDMC1B were used to rescue a defective *Saccharomyces cerevisiae* Δ rad51 strain under genotoxic conditions and data is described.

The data described here are related to the research article entitled “Characterization of recombinase DMC1B and its functional role as Rad51 in DNA damage repair in *Giardia duodenalis* trophozoites” (Torres-Huerta et al.,) [1].

**Specifications Table**

**Table:** 

Subject area	*Biology*
More specific subject area	*Recombinases GdDMC1A and GdDMC1B bioinformatics analyses*
Type of data	*Table, image, graph*
How data was acquired	*In silico analyses: Clustal W program was used to carry out protein sequence alignments*http://clustalw.ddbj.nig.ac.jp/index.php?lang=en*; ESPript server*
	http://espript.ibcp.fr/ESPript/ESPript/*was used to make Structure-based protein alignments; I-TASSER server*
	http://zhanglab.ccmb.med.umich.edu/I-TASSER/*was used for modeling protein structures*
	*Other specific methods required are also indicated in figure legends*
Data format	*Analyzed*
Experimental factors	*LD50 for ionizing radiation was calculated with G. duodenalis trophozoites*
	*Rescue of a DNA repair defective (*Δ rad51) *Saccharomyces cerevisiae* strain under DNA damage by expressing either GdDMC1A or GdDMC1B
Experimental features	*10*^*5*^*trophozoites were exposed to different doses of ionizing radiation and plotted to calculate LD50*
	*Serial dilutions of a Saccharomyces cerevisiae (wt, defective strain, defective strain transformed with either GdDMC1A or GdDMC1B bearing plasmid) in liquid culture were spotted on hydroxyurea plates or UV-treated in minimal media plates.*
Data source location	*Genetics and Molecular Biology Department. Centro de Investigación y Estudios Avanzados del I. P. N., Mexico*
Data accessibility	*Data is within this article and available via the following links:*
	*GdDMC1A from WB (A) strain*http://www.ncbi.nlm.nih.gov/protein/XP_001709425.1*, GdDMC1B from WB (A) strain*http://www.ncbi.nlm.nih.gov/protein/XP_001710001.1*;*
	*GdDMC1A from A2*http://www.ncbi.nlm.nih.gov/protein/ESU39783.1*GdDMC1B from A2*http://www.ncbi.nlm.nih.gov/protein/ESU39512.1*; GdDMC1A from B (B-GS)*http://www.ncbi.nlm.nih.gov/protein/EET00637.1*, GdDMC1B from B (B-GS)*http://www.ncbi.nlm.nih.gov/protein/EES98708.1*; GdDMC1A from B (B-GSB)*http://www.ncbi.nlm.nih.gov/protein/ESU44340.1*, GdDMCB from B (B-GSB)*http://www.ncbi.nlm.nih.gov/protein/ESU42868.1*; GdDMC1A from E*http://www.ncbi.nlm.nih.gov/protein/EFO62265.1
	*and GdDMC1B from E*http://www.ncbi.nlm.nih.gov/protein/EFO64232.1

**Value of the data**

• Bioinformatics tools allow to identify conserved domains and/or function in recombinases GdDMC1A and GdDMC1B.
• Structure-based protein alignment allows to identify conserved domains/functions in recombinases GdDMC1A and GdDMC1B with respect to other recombinases.
• Phylogenetic analyses allow to cluster these recombinases with other organisms to determine whether they are closely related.
• Comparative modeling analysis between predicted structure of GdDMC1A and GdDMC1B can identify differences in protein structure and make some predictions.

## 1 Data

**Fig. 1:**
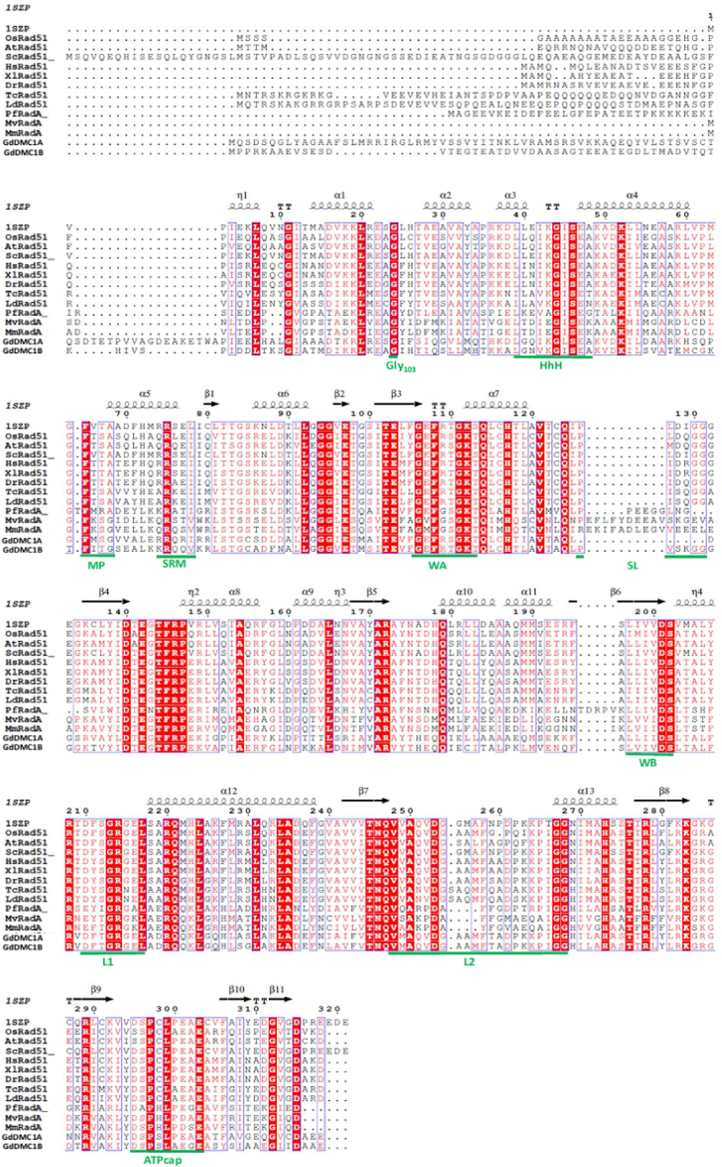
Alignment based on secondary structure of GdDMC1A and GdDMC1B against recombinases from different organisms. Sequences used are: GdDMC1A (AY295089.1) and GdDMC1B (XM_001709949), OsRad51 (*Oryza sativa* Japonica Group, NP_001066806.1), AtRad51 (*Arabidopsis thaliana*, NP_568402.1), ScRad51 (*Saccharomyces cerevisiae*, NP_011021.3), HsRad51 (*Homo sapiens*, BAA02962.1), XlRad51 (*Xenopus laevis*, NP_001081236.1), DrRad51 (*Danio rerio*NP_998371.2), TcRad51 (*Trypanosoma cruzi*, AAZ94621.1), LdRad51 (*Leishmania donovani*, AAQ96331.1), PfRadA (*Pyrococcus furiosus*, WP_011013066.1), MvRadA (*Methanococcus voltae*, O73948.1), MmRadA (*Methanococcus maripaludis*, WP_011171166.1). All motifs are shown underlined with a green line: walker A (WA), walker B(WB), Loop 1 (L1), Loop 2 (L2), Nuclear Matrix Binding Site (NMTS), helix-hairpin-helix (HhH), Subunit Rotation Motif (SRM), ATPcap motif, the group of amino acids that form the Schellman loop (SL) and the aminoacidic residue Glycine 103 (Gly103). References in color: a red box, white character: indicates strict identity; a red character: similarity in a group. This Structure-based protein sequence alignment was performed with ESPript program [5].

**Fig. 2:**
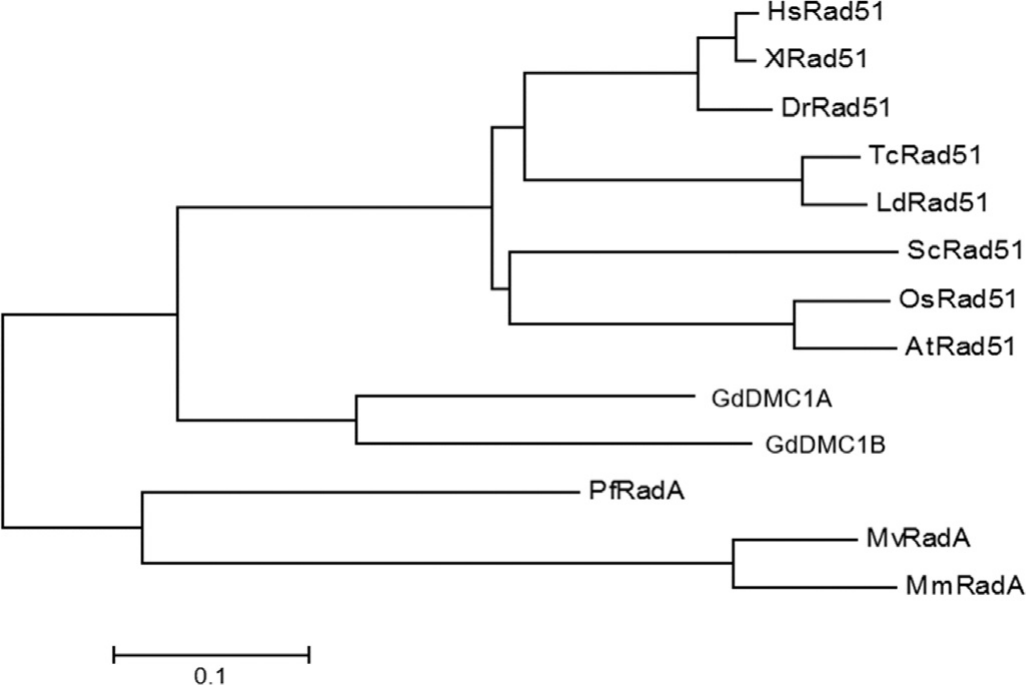
Evolutionary relationships of GdDMC1A and GdDMC1B. A phylogenetic tree was inferred using the Neighbor-Joining method [2]. The optimal tree with the sum of branch length=2.30818981 is shown. The tree is drawn to scale, with branch lengths in the same units as those of the evolutionary distances used to infer the phylogenetic tree. The evolutionary distances were computed using the Poisson correction method [3] and are in the units of the number of amino acid substitutions per site. The analysis involved 13 amino acid sequences. All positions containing gaps and missing data were eliminated. There were a total of 300 positions in the final dataset. Evolutionary analyses were conducted as described previously [4].

**Fig. 3:**
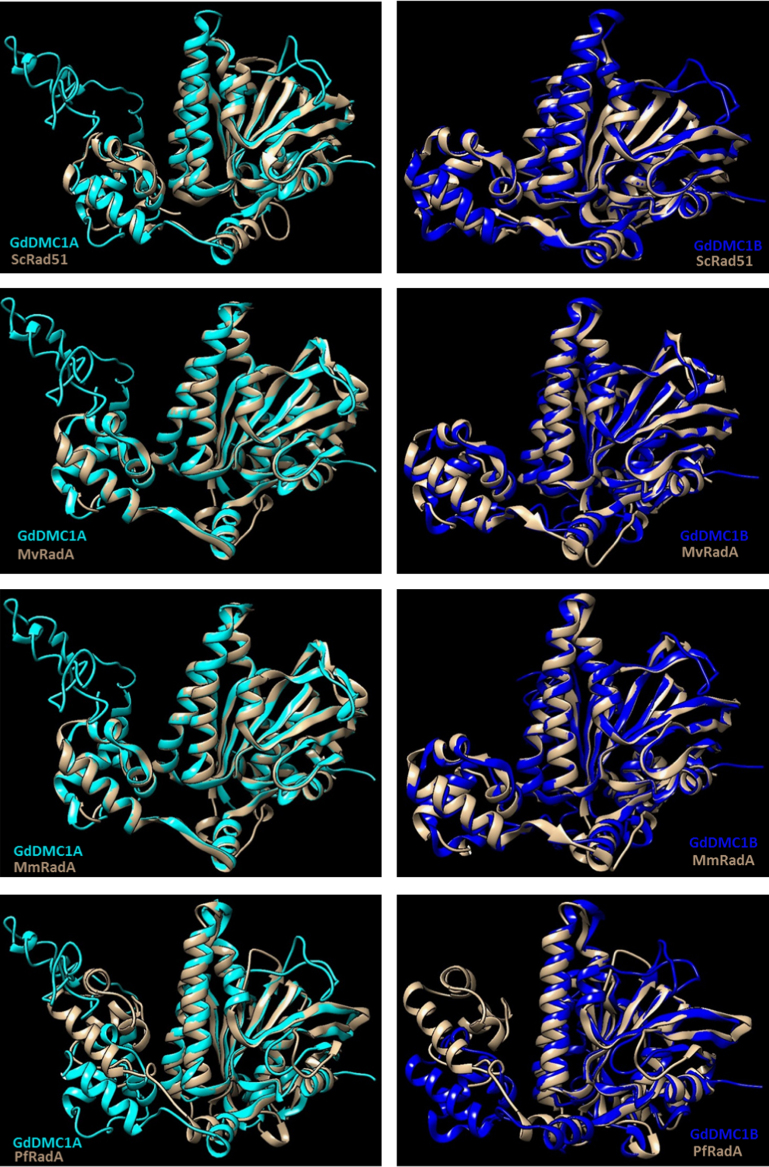
Comparison between Giardial recombinases (GdDMC1A and GdDMC1B) and other proteins that have been crystallized previously. The structures used were ScRad51 from *Saccharomyces cerevisiae* (1szpA), MvRaDA from *Methanococcus voltae* (1xu4A), MmRaDA from *Methanococcus maripaludis* (3etlA) and PfRadA from *Pyrococcus furiosus* (1pznA). GdDMC1A is shown in cyan and GdDMC1B in the darker blue. The other proteins are in light brown. Images were obtained by Chimera 1.1 software.

**Fig. 4:**
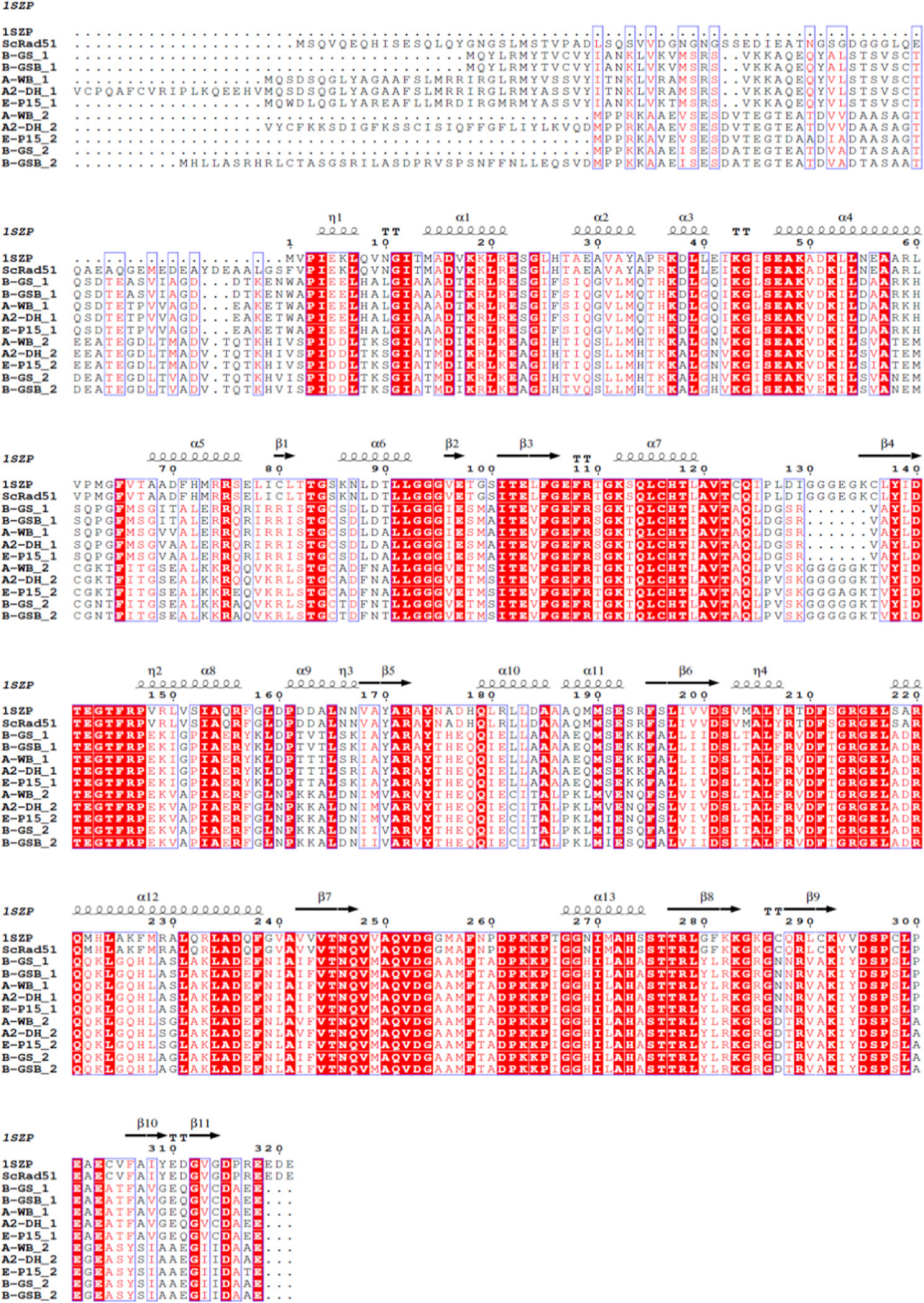
Alignment of GdDMC1A and GdDMC1B (indicated in the name for each sequence as 1 or 2 respectively) from different assemblages of *Giardia duodenalis* based on secondary structure of ScRad51 (1SZP). Sequences used are described in Table 1. Colors are as indicated in Fig. 1. This Structure-based protein sequence alignment was performed with ESPript program [5].

**Fig. 5:**
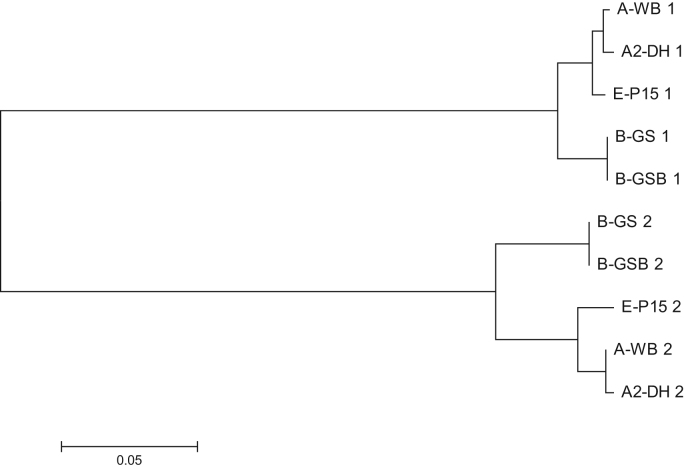
Evolutionary relationships of GdDMC1A and GdDMC1B among assemblages of *Giardia duodenalis*. The evolutionary history was inferred using the Neighbor-Joining method [2]. The optimal tree with the sum of branch length=0.52279652 is shown. The tree is drawn to scale, with branch lengths in the same units as those of the evolutionary distances used to infer the phylogenetic tree. The evolutionary distances were computed using the Poisson correction method [3] and are in the units of the number of amino acid substitutions per site. The analysis involved 10 amino acid sequences. All positions containing gaps and missing data were eliminated. There were a total of 348 positions in the final dataset. Evolutionary analyses were conducted as previously described [4]. As in Table 1, 1 refers to A and 2 refers to B.

**Fig. 6:**
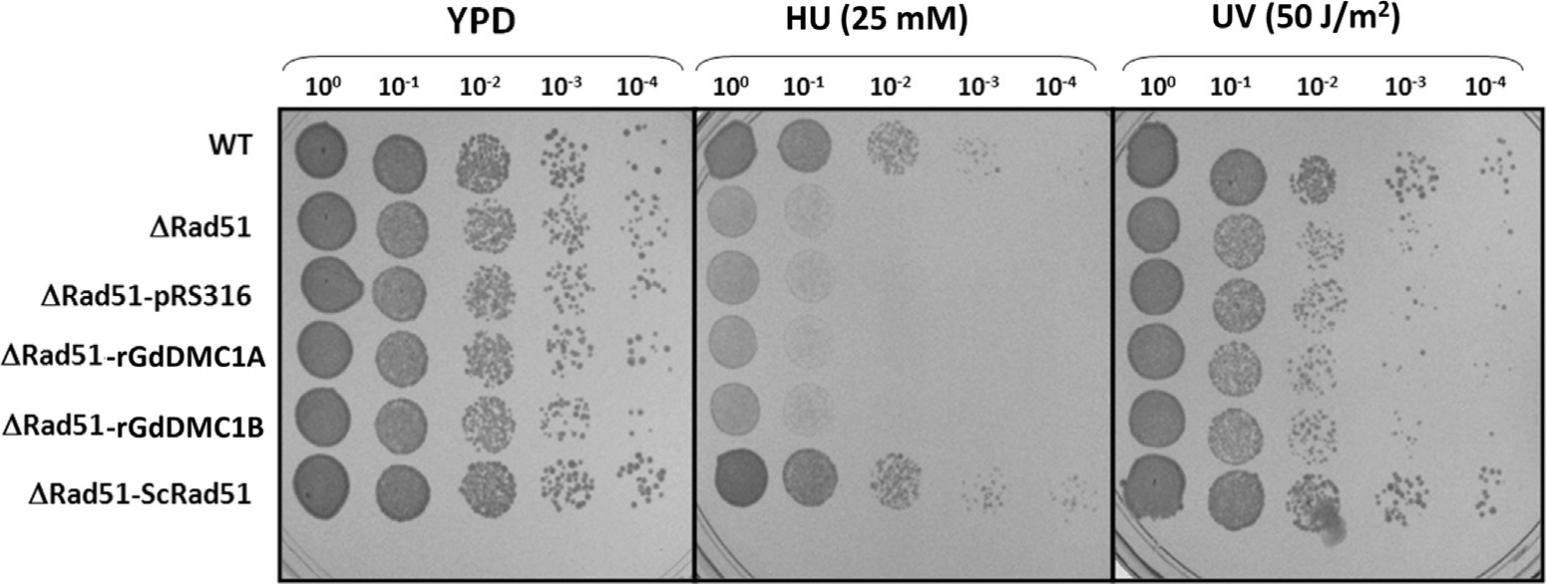
rGdDMC1A and rGdDMC1B do not complement a rad51 defective *Saccharomyces cerevisiae*. GdDMC1A and GdDMC1B were cloned in pRS316 vector, then they were transfected into a Δrad51 *Saccharomyces cerevisiae*. The ability to rescue Rad51 mutation in transfected yeast was evaluated. DNA was damaged with genotoxic agents (25 mM hydroxyurea and 50 J/m^2^ UV light). Serial dilutions were spotted into YPD plates containing the genotoxic agent and yeast growth was observed. A wild type (WT, 3595) strain was used to compare with its corresponding Δrad51 strain and the transfected yeasts with Giardial recombinases. As a positive control ScRad51 was used to complement Δrad51.

**Fig. 7:**
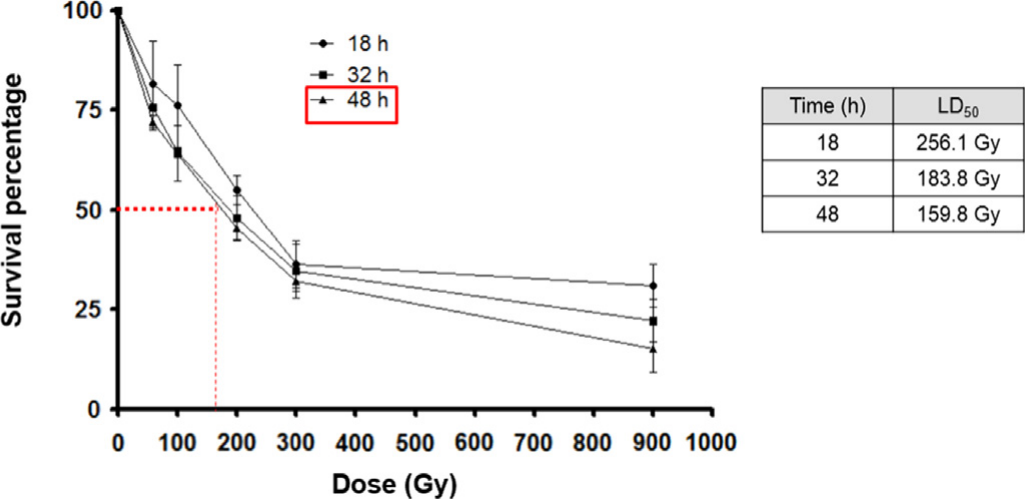
LD_50_ calculated by effect of ionizing radiation. Survival percentage of irradiated trophozoites was plotted against several dose used to treat trophozoites. Viable cells were counted with trypan blue at different times: 18, 32 and 48 h post-irradiation and LD50 was obtained with GraphPad Prism software.

Recombinases GdDMC1A and GdDMC1B amino acid sequences were compared with recombinases from other organisms (Structure-based sequence alignments) and phylogenetic reconstructions and 3D modeling were made to identify similarity (Fig. 1, Fig. 2, Fig. 3). Also, GdDMC1A and GdDMC1B protein sequences from other assemblages within *Giardia duodenalis* were identified (Table 1) and compared among them (Fig. 4, Fig. 5). Finally, GdDMC1A and GdDMC1B were expressed in a rad51 defective yeast strain (Fig. 6) and LD50 using ionizing radiation was calculated in *G. duodenalis* trophozoites (Fig. 7).

## 2 Experimental design, materials and methods

Bioinformatic tools (Structure-based sequence alignments and phylogenetic reconstructions) were used to create Fig. 1, Fig. 2, Fig. 3, Fig. 4, Fig. 5. Fig. 6 shows transforming a rad51 defective *Saccharomyces cerevisiae* strain with either GdDMC1A or GdDMC1B containing plasmids looking for a rescue phenotype under DNA damaging conditions and Fig. 7 is generated by determining LD50 at 12, 32 y 48 h after ionizing radiation exposure in *G. duodenalis*. This experimental design was used only for this work.

## Figures and Tables

**Table 1:** Data of recombinases from different assemblages of *Giardia duodenalis*. In the sequence name the first letter (A, A2, B, E) indicates the assemblage, after the hyphen the isolate of Giardia is shown and the last number 1 or 2 corresponds to proteins GdDMC1A or GdDMC1B respectively.

**Sequence name**	**Protein**	**Assemblage**	**Strain**	**Size (aa)**	**Genbank**

A-WB_1	DMC1A	A	WB C6	389	XP_001709425.1
A-WB_2	DMC1B	A	WB	368	XP_001710001.1
A2-DH_1	DMC1A	A2	DH	407	ESU39783.1
A2-DH_2	DMC1B	A2	DH	399	ESU39512.1
B-GS_1	DMC1A	B	GS/M clone H7	370	EET00637.1
B-GS_2	DMC1B	B	GS/M clone H7	368	EES98708.1
B-GSB_1	DMC1A	B	GS_B_0226	370	ESU44340.1
B-GSB_2	DMC1B	B	GS_B_0226	407	ESU42868.1
E-P15_1	DMC1A	E	P15	389	EFO62265.1
E-P15_2	DMC1B	E	P15	368	EFO64232.1

## References

[1] Torres-Huerta A.L., Martínez-Miguel R.M., Bazán-Tejeda M.L., Bermúdez-Cruz R.M. (2016). Characterization of recombinase DMC1B and its functional role as Rad51 in DNA damage repair in *Giardia duodenalis* trophozoites. Biochimie.

[2] Saitou N., Nei M. (1987). The neighbor-joining method: a new method for reconstructing phylogenetic trees. Mol. Biol. Evol..

[3] Zuckerkandl E., Pauling L., Bryson V., Vogel H.J. (1965). Evolutionary divergence and convergence in proteins. Evolving Genes and Proteins.

[4] Tamura K., Stecher G., Peterson D., Filipski A., Kumar S. (2013). MEGA6: molecular evolutionary genetics analysis version 6.0. Mol. Biol. Evol..

[5] Gouet P., Robert X., Courcelle E. (2003). ESPript/ENDscript: extracting and rendering sequence and 3D information from atomic structures of proteins. Nucleic Acids Res..

