# Supplementary material for: In silico data analyses of recombinases GdDMC1A and GdDMC1B from Giardia duodenalis
Source: Data Brief. 2016 Aug 22;9:236–44. doi: 10.1016/j.dib.2016.08.031 (PMC5021851; doi:10.1016/j.dib.2016.08.031)
Supplement: Supplementary file 1 — Supplementary material [file mmc1.pdf]

12 july, 2016

DIB

We would like to submit revision of the manuscript entitled “**In silico data analyses of recombinases GdDMC1A and GdDMC1B from *Giardia duodenalis***” assigned with number DIB-D-16-00419 by Ana Laura Torres-Huerta, Rosa María Martinez-Miguel, María Luisa Bazán-Tejeda, & Rosa María Bermúdez-Cruz and declare that the material submitted is original, that all authors are in agreement to have the article published and that there is no conflict of interest.

Best regards

Dr. Rosa Maria Bermudez-Cruz
Professor of the Department of Genetics and
Molecular Biology
CINVESTAV-IPN
Mexico, DF. Mexico
Phone: + 52 5557473800 xt 5335
e-mail: roberm@cinvestav.mx
